# Flexible Bronchoscopy for Sewing Pin Removal From Intrabronchial in a Young Adult: A Case Report From Calmette Hospital, Cambodia

**DOI:** 10.1002/rcr2.70402

**Published:** 2025-11-05

**Authors:** Menghak Heng, Bunleng Kou, Saly Saint, Ing Cheng, Thy Try, Pathy Ngeth, Bunpaul Chhar

**Affiliations:** ^1^ Pulmonology Unit, Medical Ward ‘A’ Calmette Hospital Phnom Penh Cambodia; ^2^ Faculty of Medicine University of Health Sciences Phnom Penh Cambodia; ^3^ Department of Neurosurgery Calmette Hospital Phnom Penh Cambodia

**Keywords:** aspiration, Cambodia, flexible bronchoscopy, intrabronchial, sewing pin

## Abstract

Foreign body aspiration is a frightening experience that can be life‐threatening. Although it occurs more frequently in children, it can also affect adults, often related to neurological illness, dental procedures, or accidental aspiration during daily activities. Diagnosis relies on clinical history, imaging and bronchoscopy. We report a case of a 20‐year‐old Cambodian man, a wedding decorator, who accidentally inhaled a sewing pin while laughing during work. He presented with persistent cough and dyspnoea. Chest radiography initially showed the pin in the trachea, which later migrated into the left main bronchus. The foreign body was successfully removed using flexible bronchoscopy under moderate sedation, after local anaesthesia proved inadequate. The patient was discharged the same day without complications. This case demonstrates the effectiveness of flexible bronchoscopy and the importance of timely intervention in preventing surgical intervention, particularly in resource‐limited settings.

## Graphical abstract

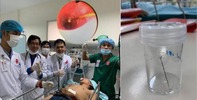

## 1 Introduction

In the last decade, foreign body aspiration has been reported more frequently both in the local population and globally. It is more common in children, less common in adults [1, 2, 3, 4] and can cause sudden distress or life‐threatening complications. Risk factors in adults are related to neurological illness affecting swallowing, alcohol consumption, dental procedures and sudden inspiration during talking or laughing [1, 2].

Because symptoms can be nonspecific and chest radiographs occasionally normal, clinical suspicion must remain high [4]. Flexible bronchoscopy remains an essential diagnostic and therapeutic tool for airway foreign bodies [1, 5]. Surgical intervention is reserved for cases where bronchoscopic extraction fails or is contraindicated [1].

We report a case of sewing‐pin aspiration in a young Cambodian adult successfully managed with flexible bronchoscopy by a compassionate and coordinated multidisciplinary team. The case underscores how a simple household object can become life‐threatening and highlights the growing importance of bronchoscopy services in Cambodian hospitals.

## 2 Case Report

A 20‐year‐old male wedding decorator with no medical history presented to Calmette Hospital with acute cough and dyspnoea after accidentally inhaling a sewing pin while laughing at work. The patient and his family were distressed, fearing long‐term consequences for his health and livelihood.

**FIGURE 1:**
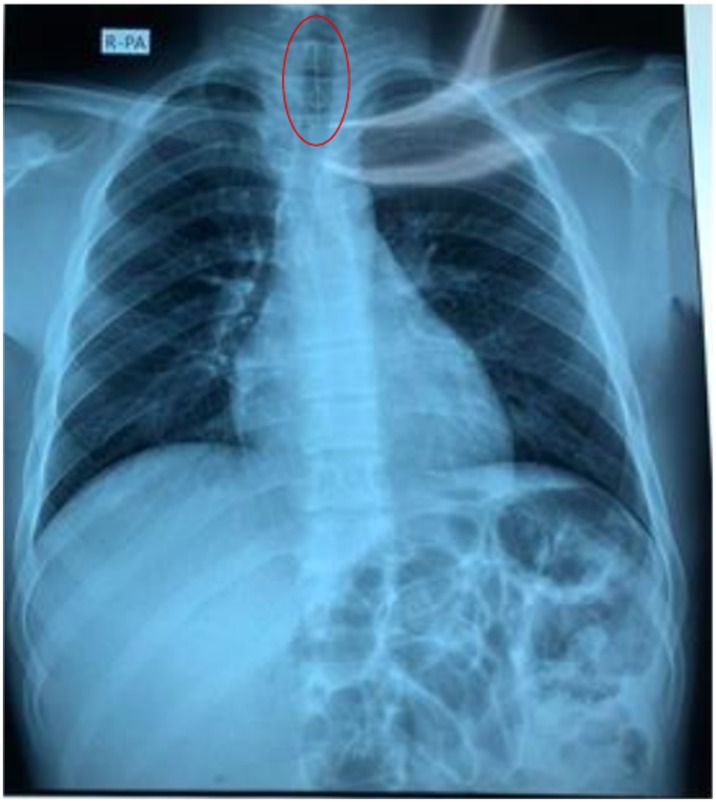
Chest x‐ray shows the sewing pin in the trachea.

**FIGURE 2:**
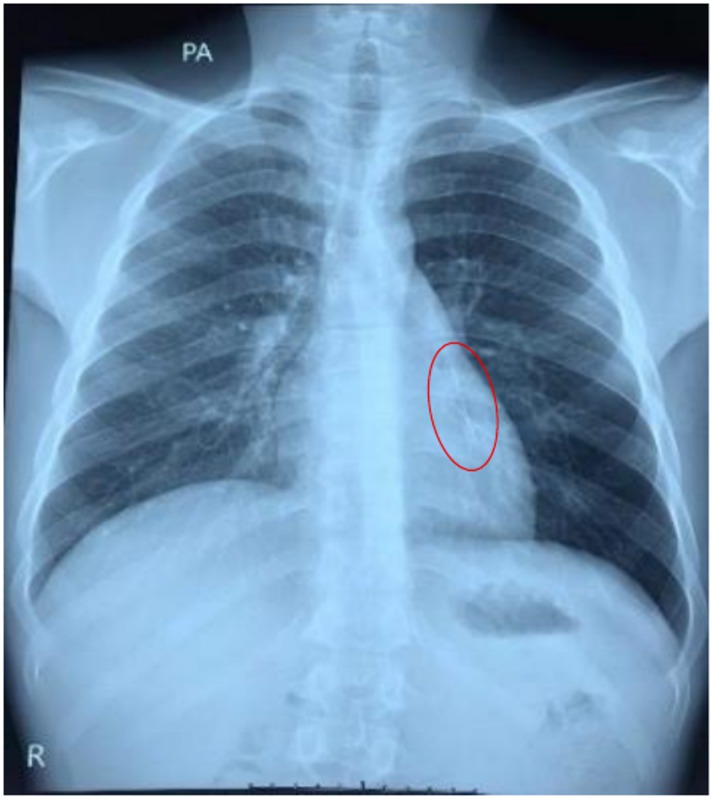
Chest x‐ray demonstrates migration of the sewing pin to the left main bronchus.

Initial chest x‐ray (Figure 1) revealed the pin lodged in the trachea, which later migrated into the left main bronchus (Figure 2). Flexible bronchoscopy was conducted by a team of pulmonologists under moderate sedation with anaesthesiologist support as local anaesthesia alone was not tolerated by the patient.

**FIGURE 3:**
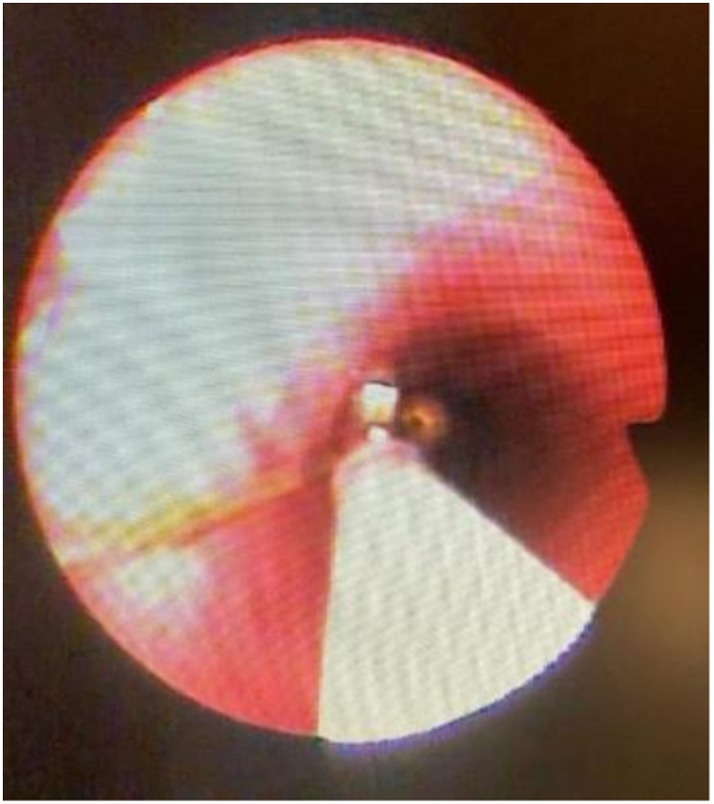
Bronchoscopy view of the sewing pin lodged on the main left‐sided intrabronchial.

**FIGURE 4:**
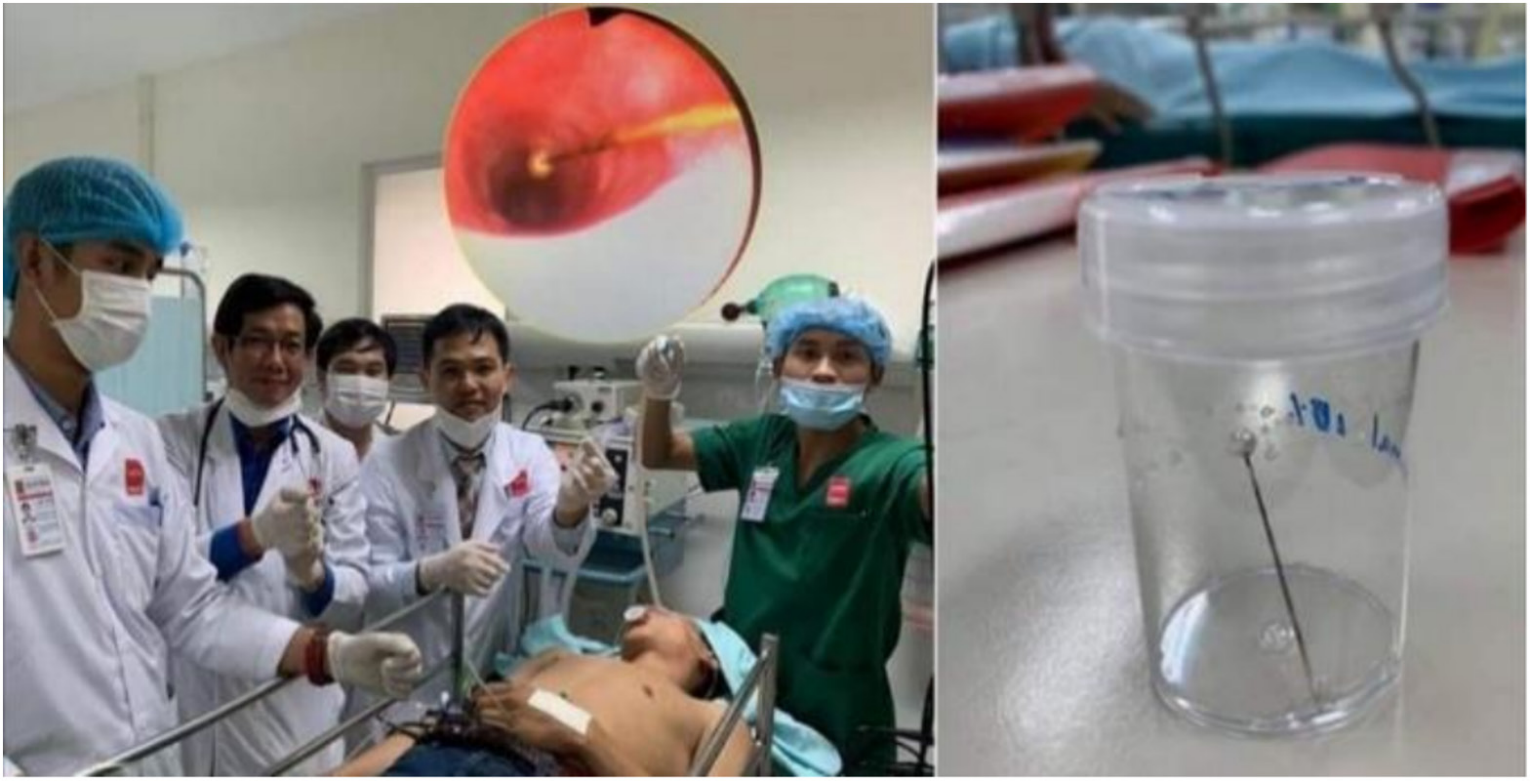
Extraction of the pin by using flexible bronchoscopy by teamwork.

The pin was visualised in the left main bronchus (Figure 3) and carefully grasped at its proximal end. The extraction was technically challenging because of limited visualisation and risk of mucosal injury, requiring multiple careful repositioning. After approximately 20 min, the pin was removed successfully (Figure 4). Minor bronchial mucosal oedema and slight bleeding were observed but no perforation or major complication occurred. The patient recovered uneventfully and was discharged the same day. Three‐month follow‐up showed no recurrence or late sequelae.

## 3 Discussion

Foreign body aspiration in adults is rare but can lead to severe respiratory complications [1, 2, 3]. Adults at risk include those with neurological impairment, altered consciousness, or dental procedures, but it can also occur during laughter or conversation [2, 3], as in this case. Elderly individuals are more prone to inhaling foreign bodies while sleeping due to inadequate toothbrushing, a weakened cough reflex and difficulty swallowing [1]. Foreign objects frequently encountered include peanuts, sunflower seeds, coins or metallic objects [1].

Typical symptoms include coughing, suffocation episodes and sometimes cyanosis [1]. Untreated aspiration can result in pleurisy, pneumonia, lung abscess, pneumothorax, or obstructive emphysema [1].

Medical history, physical examination and x‐rays are important for diagnosis; a normal clinical exam does not rule out the absence of a foreign object in the airways [1, 2, 3]. Clinical suspicion should remain high even when physical findings are inconclusive [1, 2]. Radiographic evaluation can help localise metallic objects [3, 4, 5], though migration is possible, as illustrated in this case.

Bronchoscopy remains the cornerstone for diagnosis and treatment of airway foreign bodies [2, 4]. While rigid bronchoscopy is traditionally used [4], flexible bronchoscopy offers a less invasive approach that can be performed successfully in adult patients [1, 2, 3, 5]. It provides a minimally invasive and effective alternative in adults, particularly when performed by experienced operators [2, 3, 5]. There is no consensus on the use of rigid or flexible bronchoscopy; the choice depends on the experience and skill of performing with each endoscope [4, 5]. In this case, moderate sedation ensured airway stability and patient cooperation, enabling a successful procedure without surgical intervention. The teamwork among pulmonologists and anaesthesiologists was crucial in ensuring safety and efficiency.

This case illustrates how a moment of laughter in daily work can lead to a life‐threatening event. In Cambodia, where young adults are central to family income, provision of timely and safe medical care has profound social value. This case conveys three key messages: (i) early imaging and high clinical suspicion are vital for diagnosis; (ii) flexible bronchoscopy is used for both diagnostic and therapeutic purposes and can safely prevent thoracotomy; and (iii) effective multidisciplinary collaboration is the key to success, especially in resource‐limited environments.

In conclusion, flexible bronchoscopy serves as the primary diagnostic and therapeutic modality in case of diagnostic uncertainty. It proves to be a safe, effective and minimally invasive method for removing airway foreign bodies in adults. The choice between flexible and rigid techniques depends on institutional resources and operator expertise. This case highlights how early intervention and collaborative care can prevent surgical complications and reduce patient burden.

## Author Contributions

All authors have reviewed the final version to be published and agreed to be accountable for all aspects of our work.

## Consent

The authors declare that written informed consent was obtained for the publication of this manuscript and accompanying images and attest that the form used to obtain consent from the patient complies with the Journal requirements as outlined in the author guidelines.

## Conflicts of Interest

The authors declare no conflicts of interest.

## Data Availability

The data that support the findings of this study are available on request from the corresponding author. The data are not publicly available due to privacy or ethical restrictions.
